# Quality and Timing of Stressors Differentially Impact on Brain Plasticity and Neuroendocrine-Immune Function in Mice

**DOI:** 10.1155/2013/971817

**Published:** 2013-03-31

**Authors:** Sara Capoccia, Alessandra Berry, Veronica Bellisario, Davide Vacirca, Elena Ortona, Enrico Alleva, Francesca Cirulli

**Affiliations:** ^1^Section of Behavioural Neuroscience, Department of Cell Biology and Neurosciences, Istituto Superiore di Sanità, Viale Regina Elena 299, 00161 Rome, Italy; ^2^Section of Biomarkers in Degenerative Diseases, Department of Cell Biology and Neurosciences, Istituto Superiore di Sanità, Viale Regina Elena 299, 00161 Rome, Italy

## Abstract

A growing body of evidence suggests that psychological stress is a major risk factor for psychiatric disorders. The basic mechanisms are still under investigation but involve changes in neuroendocrine-immune interactions, ultimately affecting brain plasticity. In this study we characterized central and peripheral effects of different stressors, applied for different time lengths, in adult male C57BL/6J mice. We compared the effects of repeated (7 versus 21 days) restraint stress (RS) and chronic disruption of social hierarchy (SS) on neuroendocrine (corticosterone) and immune function (cytokines and splenic apoptosis) and on a marker of brain plasticity (brain-derived neurotrophic factor, BDNF ). Neuroendocrine activation did not differ between SS and control subjects; by contrast, the RS group showed a strong neuroendocrine response characterized by a specific time-dependent profile. Immune function and hippocampal BDNF levels were inversely related to hypothalamic-pituitary-adrenal axis activation. These data show a fine modulation of the crosstalk between central and peripheral pathways of adaptation and plasticity and suggest that the length of stress exposure is crucial to determine its final outcome on health or disease.

## 1. Introduction

Stressful events are well-known risk factors that can promote neurochemical changes ultimately involved in the pathophysiology of psychiatric disorders such as major depression [1–3]. Any change of the internal or external milieu may represent a source of stress triggering a complex and coordinated set of physiological responses involving (among others) the activation of the hypothalamus-pituitary-adrenal (HPA) axis [4–6]. Although adaptive on the short run, prolonged exposure to glucocorticoids hormones (GC), secreted following stress, may exhaust the capacity of an organism to cope with further stressors and, given the catabolic nature of these adrenal glucocorticoids, lead to an impairment in brain plasticity [5, 7, 8]. 

Stress begins in the brain with the perception and interpretation of the stressful event and affects the brain itself as well as the rest of the body through plastic changes, leading to adaptation. The connection between central stress response pathways and peripheral targets involves the alteration of a number of neurochemical and/or inflammatory factors that ultimately affect neuronal functioning and/or survival [8, 9]. One of the most representative players implicated in these events is the neurotrophin brain-derived neurotrophic factor (BDNF), which is involved in synaptic and morphological plasticity of the brain both during development (with maximal levels during times of neuronal growth, differentiation and synaptogenesis) as well as at adulthood [10–13]. High levels of this neurotrophin are found in the hippocampus, a brain region expressing also high levels of receptors for GC (GR) and playing a main role in the negative feedback regulation of the HPA axis, a pathway often disinhibited in depressed subjects [14]. A growing body of evidence shows that chronic stress decreases the expression of BDNF contributing to neuronal atrophy in the hippocampus and that antidepressant treatment reverses or blocks these effects, restoring brain plasticity [8, 9, 15, 16]. 

By being able to directly affect HPA axis activity [9, 17] and being produced by cells outside the nervous system (including immune cells, adipocytes, endocrine, and endothelial cells), BDNF has a key position in integrating neural, immune, and endocrine responses to stress [8, 18, 19]. Indeed, the central nervous system and the immune system are known to be engaged in an intense bidirectional crosstalk which can be affected by stress and which involves multiple mediators, including cytokines and growth factors [20]. As an example, the immune signaling cytokines, particularly the proinflammatory ones such as interleukin-6 (IL-6) or tumor necrosis factor-alpha (TNF-*α*), are elevated following stress exposure and can thwart brain plasticity eliciting depressive symptoms, which are amenable to antidepressant treatment [20]. However, the directionality of the effects of stress is still a matter of intense investigation: for instance, GC released in response to stress can act both enhancing and inhibiting immune responses and by decreasing or increasing levels of neurotrophins [21–24]. Such opposite effects might coexist in light of the fact that during stress, multiple interacting mediators are activated in a nonlinear network influencing different systems and functions [25]. Factors such as the duration (acute versus chronic) of stress as well as the time of exposure to GC, relative to the activation and time course of the immune response, might differently impact health outcome [21]. Progress in understanding the pathophysiology of stress would greatly benefit from further preclinical studies incorporating both the permissive as well as the inhibitory role of GC in immune-endocrine interactions and mimic conditions experienced in everyday life [26].

Restraint stress (RS) and the chronic disruption of the social hierarchy (SS) are two of the most widely used experimental paradigms that can induce stress in mice. The first relies on a combination of psychological and physical stimuli and is considered a reliable model of severe stress in humans [27, 28]; the latter represents a comprehensive and ethologically relevant paradigm inducing chronic stress and leading to anxiety and/or depressive-like symptoms as often reported in stress-precipitated major depression [29–31]. Thus, the main aim of the present study was to characterize central and peripheral effects of different stressors, applied for different time lengths on neuroendocrine and immune responses in adult male C57BL/6J mice. Specifically, we compared the effects of repeated (7 versus 21 days) RS and SS on neuroendocrine (circulating corticosterone) and immune (circulating cytokines and splenic apoptosis) function and on a marker of brain plasticity (hippocampal BDNF) in order to identify a specific neuroendocrine profile in response to a selective type of stress.

## 2. Materials and Methods

### 2.1. Animals

Experimental subjects were adult male C57BL/6J mice purchased from a commercial breeder (Charles River, Calco, Italy). Upon arrival, all animals were group-housed in the same room provided by air conditioning (temperature 21 ± 1°C, relative humidity 60 ± 10%), in transparent Plexiglas cages (29 cm × 12 cm × 14 cm), under a reversed 12/12 h light/dark cycle with lights off from 0800 to 2000 h. Pellet food (standard diet Altromin-R, Rieper, Italy) and tap water were continuously available. All stressors were administered randomly throughout the active phase of the day. A Social Interaction Test was used as a challenge to assess HPA axis response following the social stress procedure and took place between 1700–2000 h, that is, during the corticosterone (CORT) circadian trough. All subjects were sacrificed at the end of the stress procedure. Animal handling and experimental procedures were performed in accordance with the EC guidelines (EC Council Directive 86/609 1987) and with the Italian legislation on animal experimentation (Decreto L.vo 116/92).

### 2.2. Experimental Procedures

#### 2.2.1. Experiment I: Effects of Restraint Stress on Neuroendocrine and Immune Responses

Experimental subjects were 15 mice divided into three groups: 7 days restraint stress (RS7, *n* = 5), 21 days restraint stress (RS21, *n* = 5), and unhandled controls (CTRL, 5 subjects left undisturbed in their home cage). All subjects undergoing the same treatment condition were group-housed. The restraint procedure consisted in removing subjects from their home cage and putting each of them in a conical 50 mL falcon tube, provided with holes for breathing, on a laboratory bench under dim light for 3 consecutive hrs/day. The stress was administered each day at random times in order to prevent habituation to the procedure. Animals from the RS21 were used to assess stress-related changes in CORT levels so to have repeated measures for each subject during days 1, 7, and 21. On these days the procedure was administered at a fixed times in order to take into account circadian rhythm, that is, from 1700 to 2000. Blood samples were collected by tail nick at 0 (basal) and 180 min from the onset of stress (i.e., at 2000). At the end of stress all mice (CTRL, RS7, and RS21) were sacrificed, trunk blood was collected to assess levels of the proinflammatory cytokines interleukin 6 (IL-6) and tumor necrosis factor-alpha (TNF-*α*), and of the anti-inflammatory cytokine Interleukin 10 (IL-10), [32, 33]. Brain and spleen were dissected out in order to assess, respectively, hippocampal BDNF levels and lymphocyte apoptosis.

#### 2.2.2. Experiment II: Effects of Social Stress on Neuroendocrine and Immune Responses

Experimental subjects were 48 adult male mice divided into three groups: 7 days social stress (SS7, *n* = 16), 21 days social stress (SS21, *n* = 16), and controls (CTRL, 16 subjects group-housed). All mice undergoing the SS procedure were ear-marked and housed into 4 cages (4 mice/cage) and social structure was disrupted twice a week for one or three weeks by replacing one mouse with a novel unfamiliar selected randomly from another cage [34, 35]. Sawdust was replaced at the same time in all cages. Control mice were also ear-marked and housed in stable groups of 4 mice/cage. Cages were cleaned and sawdust replaced twice a week mimicking the handling procedure of the SS groups [31]. 

The activity of the HPA axis was assessed in response to a 20-minute acute stress (Social Interaction Test) and blood samples for CORT evaluation were collected from 8 subjects per group (CTRL, SS7, SS21) right before (basal) and 30 min following the end of stress. Briefly, the night before the Social Interaction Test, all subjects were individually housed to stimulate social interactions [36–38]. On the day of test, mice were placed in a novel cage, identical to the holding cage, ideally subdivided in three equal parts, with an unfamiliar conspecific of the same strain, weight, and sex that had been previously isolated (standard opponent). Standard opponents were marked with a yellow, scentless, and nontoxic paint [31].

At the end of the test mice that did not undergo the Social Interaction test (5 mice for each group, randomly chosen) were sacrificed, and trunk blood was collected to assess also levels of IL-6, TNF-*α*, and IL-10 [32, 33]. Brains and spleen were dissected out, in order to assess, respectively, hippocampal BDNF levels and lymphocyte apoptosis.

### 2.3. Radioimmunoassay for Corticosterone Determination-RIA

Blood samples (100 *μ*L, approximate volume) were collected individually in potassium EDTA coated tubes (1.6 mg EDTA/mL blood, Sarstedt, Germany). All samples were kept on ice and later centrifuged at 3000 rpm for 15 min at +4°C. Blood plasma was transferred to Eppendorf tubes for CORT determination and stored at −20°C until further analysis. CORT was measured using a commercially available radioimmunoassay (RIA) kit containing 125Iodine-labeled CORT; 5 *μ*L of plasma was sufficient to carry out CORT measurement. Sensitivity of the assay was 0.125 mg/dL, inter- and intra-assay variation was less than 10 and 5%, respectively (MP Biomedicals Inc., CA, USA). Vials were counted for 2 min in a gamma-scintillation counter (Packard Minaxi Gamma counter, Series 5000).

### 2.4. BDNF Measurement

BDNF evaluation was carried out with an enzyme-linked immunosorbent assay kit (BDNF Emax ImmunoAssay System number G7610, Promega, Madison, Wisconsin, USA) following the instructions provided by the manufacturer. Following sacrifice brains were quickly removed and the hippocampus was dissected out and immediately stored at −80°C until used. Brain tissues were homogenized in a lysis buffer and centrifuged at 14000 rpm, and the supernatant was used for BDNF analyses. Briefly, BDNF standard and brain samples were distributed in 96-well immunoplates precoated with monoclonal anti-mouse BDNF antibody (100 mL/well) and incubated for 2 h at room temperature. After washing, plates were incubated with an anti-human BDNF antibody for 2 h at room temperature. The plates were washed again and then incubated with an anti-IgY horseradish peroxidase (HRP) for 1 h at room temperature. Tetramethylbenzidine (TMB)/peroxidase substrate solution was added to the wells to produce colorimetric reaction measured at 450 nm with a microplate reader (Dynatech MR 5000, Dynatech Laboratories, Chantilly, VA, USA). BDNF concentrations were determined from the regression line for the BDNF standard incubated under similar conditions in each assay. The sensitivity of the assay was about 15 pg/mg of BDNF, and the cross-reactivity with other related neurotrophic factors (NGF, NT-3, and NT-4) is considered nil [39].

### 2.5. Cytokines Determination

Quantitative evaluation of TNF-*α*, IL-6, and IL-10 in sera from trunk blood of stressed and control mice was determined by ELISA kits (R&D Systems, Inc., Minneapolis, USA) according to the manufacturer's instructions. Briefly, standards, controls, and sera were placed into the wells and incubated 2 h at room temperature. After washing 5 times, the enzyme-linked polyclonal antibody specific for mouse cytokines was added to the wells and then, after washing, the substrate solution was added. The enzyme reaction was read at 450 nm (correction wavelength set at 570 nm). The samples values were read off the standard value.

### 2.6. Splenocytes Apoptosis

Spleens were gently removed and suspended in ice-cold culture RPMI-1640 medium (GIBCO BRL, Grand Island, NY). Splenocytes were isolated from mice spleen by flushing 5 mL of RPMI-1640 medium into spleen by needle and syringe. Cells were then centrifugated at 1200 rpm in order to remove cellular debris. Cells were resuspended in supplemented RPMI 1640 and counted on a hemocytometer in trypan blue to ensure viability. Average viability was >90%. Splenocytes were then cultured in RPMI-1640 medium with 10% FBS (Euroclone, Pero, Italy), 2 mM glutamine (Sigma, St Louis, MO), and 50 *μ*g/mL gentamycin (Sigma). Apoptosis was measured after 1 h of culture. Apoptosis was quantified using FITC-conjugated annexin V (AV) and propidium iodide (PI) apoptosis detection kit (Marine Biological Laboratory, Woods Hole, MA) according to the manufacturer's protocol. Reported data are referred to AV-positive apoptotic cells. AV binds to phosphatidylserine which is exposed at the outer surface of the cell membrane already at early stages of apoptosis and remains so during the subsequent process of apoptosis. By defining apoptotic cells as those cells staining with AV, irrespective of PI staining, we were able to detect early (AV+/PI− cells) as well as late (AV+/PI+ cells) apoptotic cells. In this study, we analyzed specifically “early apoptosis” in which the nuclear changes are observed first, in contrast to the changes seen in the later stages of apoptosis and then in the necrosis, which usually begin with cell membrane damage [40, 41]. Acquisition was performed on a FACSCalibur cytometer (BD Immunocytometry Systems) and 50.000 events per sample were run. Data were analyzed using the Cell Quest Pro (BD Immunocytometry Systems) software. 

### 2.7. Statistical Analysis

Data were analyzed using parametric analysis of variance (ANOVA) with “condition” (control and stress) as between-subjects factor (BDNF, cytokines, apoptosis, CORT) and “day” (1, 7, 21) and “time” (0 and 180) as within-subject repeated measures (CORT assessment only for the restraint stress). *Post hoc* comparisons were performed using the Tukey's test. Statistical analysis was performed using Statview II (Abacus Concepts, CA, USA). Data are expressed as mean + SEM. A significance level of 0.05 was chosen.

## 3. Results

### 3.1. Experiment I: Restraint Stress

#### 3.1.1. Corticosterone

**Figure 1:**
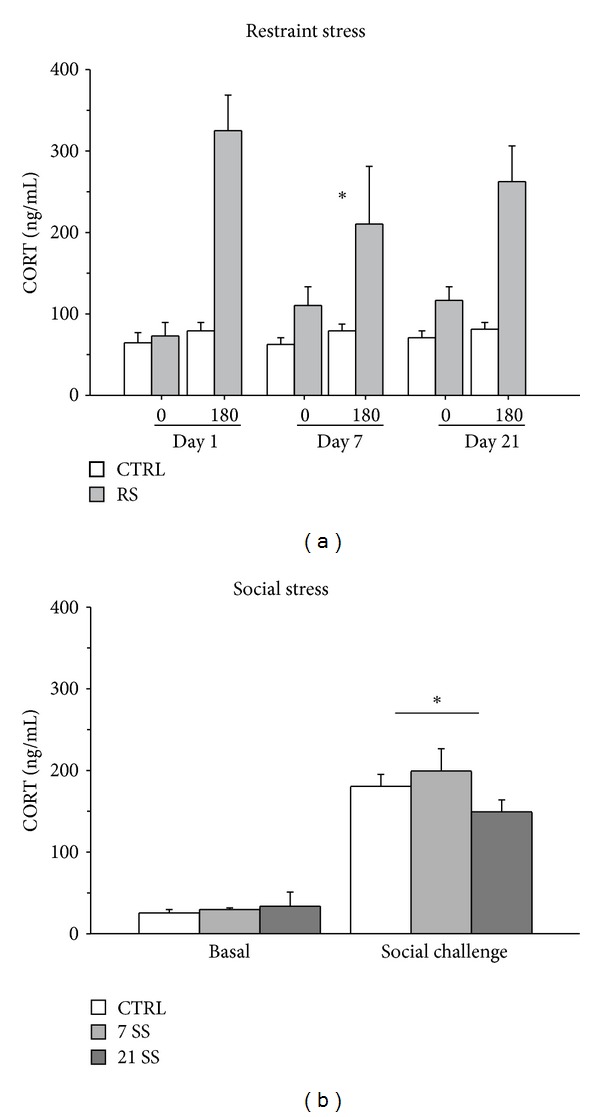
Effect of restraint stress on CORT secretion in mice. All subjects undergoing RS showed a reduced response of the HPA axis on day 7 (a). Effect of social stress on CORT secretion in mice. The response to an acute challenge (represented by the Social Interaction Test) was effective in inducing an increase in CORT secretion in all groups, with no differences in relation to social stress exposure (b). Results are presented as mean + S.E.M. *P < 0.05.

Restraint stress was effective in challenging the HPA axis. In fact, RS subjects showed overall higher CORT levels (main effect of condition: *F*(1,8) = 17.917, *P* = 0.0029) compared to controls, particularly 180 minutes from the onset of stress (interaction between condition and time: *F*(1,8) = 12.022, *P* = 0.0085). Moreover, a blunted HPA axis response characterized RS subjects on day 7 (main effect of days *F*(1,8) = 3.618 *P* = 0.0505; see Figure 1(a)).

#### 3.1.2. BDNF

BDNF evaluation was performed on 4 mice in each group since values from some subjects (1 subject for each group) were found to be outliers and were therefore discarded from the analysis (Grubbs' test performed by GraphPad Software). 

**Figure 2:**
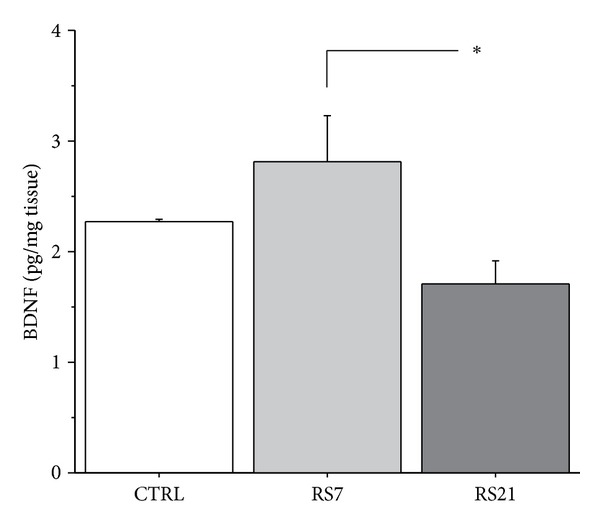
Effects of restraint stress on hippocampal BDNF levels. BDNF levels were decreased following a chronic 21 days restraint procedure compared to 7 days of repeated restraint. Data shown are mean + S.E.M. *P < 0.05.

A time-dependent effect of RS was found for hippocampal BDNF levels (see Figure 2). In particular, *post hoc* comparisons show a decrease in BDNF levels following 21 days of RS compared to RS7 (main effect of condition *F*(2,9) = 4.164, *P* = 0.0500). The latter group did not differ from the CTRL subjects. However, it is worth noticing that the lack of difference between these two groups might be related to a reduced power of the statistical test (0.574) suggesting that this result suffers from a low number of experimental subjects (only 4 animals per experimental group) possibly masking other significant trends (RS7 versus CTRL). 

#### 3.1.3. Cytokine Production

**Figure 3:**
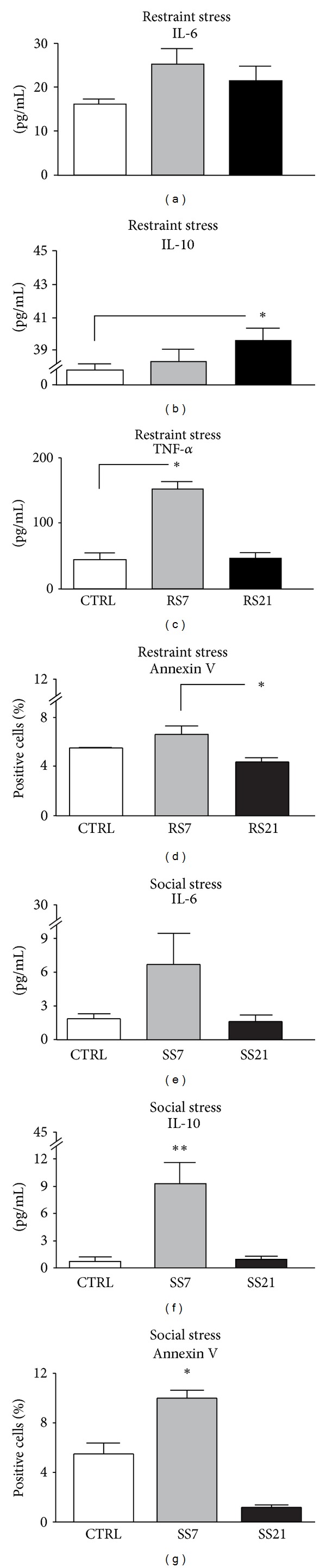
Effect of RS and SS on the immune system response. *Restraint stress procedure*. Following 7 days of restraint stress the proinflammatory cytokine TNF-*α* increases, (b) while the increase in IL-6 during days 7 and 21 just missed statistical significance (a); by contrast the anti-inflammatory cytokine IL-10 increased following 21 days (c). The percentage of apoptotic splenocytes was found to be decreased following 21 days of stress (d). *Social stress procedure*. Levels of IL-10 were increased already after 7 days of the SS procedure (f). Splenocytes apoptosis was increased after 7 days of SS and decreased following 21 days (g). No difference is evident as for levels of IL-6 (e). Results are presented as mean + S.E.M. **P < 0.01, *P < 0.05.

Following 7 days of restraint stress a tendency to increase was observed for levels of IL-6 (*F*(2,12) = 3.480; *P* = 0.0643, Figure 3(a)). This trend reached statistical significance when assessing TNF-*α* (main effect of condition: *F*(2,12) = 5.558; *P* = 0.0196, Figure 3(b)) that returned to basal levels after 21 days. By contrast, IL-10 increased only after 21 days of restraint (main effect of treatment: *F*(2,12) = 5.345; *P* = 0.0219, Figure 3(c)).

#### 3.1.4. Splenocytes Apoptosis

Splenocytes apoptosis slightly decreased after 21 days of restraint (main effect of condition: *F*(2,12) = 8.597; *P* = 0.0048, *post hoc* RS7 days versus RS21 days *P* < 0.01; see Figure 3(d)). No difference was found between CTRL and RS7. 

### 3.2. Experiment II—Social Stress

#### 3.2.1. Corticosterone

The Social Interaction Test was effective in inducing the activation of the HPA axis in all groups, regardless of their stress history (effect of social challenge: *F*(1,29) = 153,515; *P* < 0.0001, Figure 1(b)). 

#### 3.2.2. BDNF

Social stress, per se, did not affect hippocampal BDNF levels (no main effect of condition: *F*(2,12) = 2.015 *P* = 0.1759, data not shown). 

#### 3.2.3. Cytokine Production

Social condition did not affect significantly the production of IL-6, even if a slight increase after 7 days of social stress was observed, (effect of condition: *F*(2,12) = 3.075 *P* = 0.0835, see Figure 3(e)). By contrast, serum levels of IL-10 increased after 7 days of the social stress procedure (main effect of condition: *F*(2,12) = 13.217 *P* = 0.0009, see Figure 3(f)). Differences in serum TNF levels between control and social stressed mice appeared undetectable (data not shown).

#### 3.2.4. Splenocytes Apoptosis

Splenocytes apoptosis increased after 7 days of social stress and decreased following 21 days (main effect of treatment: *F*(2,12) = 47.932; *P* < 0.0001, see Figure 3(g)). 

## 4. Discussion

Data from this study show that chronic RS is a powerful stressor eliciting strong neuroendocrine, and immune responses and that brief *versus* prolonged exposure to this stress results in a differential activation of these systems in mice. In addition, we were able to identify a specific neuroendocrine-immune profile associated to specific changes in hippocampal BDNF levels. Results suggest a fine modulation of the crosstalk between central and peripheral pathways of adaptation and plasticity and that the length of stress exposure is crucial to determine its final outcome on health or disease.

Allostasis—or “stability through change”—is defined as any neural, neuroendocrine and immune activation leading to adaptation in the face of stressful challenges [4]. While in the short run, activation of these systems is essential to the maintenance of homeostasis and survival yet, over longer time intervals, it imposes a cost-allostatic load—that can accelerate disease processes or participate to pathological changes associated, among others, to immunosuppression [4]. When we studied the characteristics of the diverse stressors applied for different lengths of times, we found that upon prolonged exposure to RS (21 days), an increase in the immunogenic/allostatic load was observed, mirrored by a peak in CORT levels comparable to that observed on day 1. This was associated to a suppression of the immune system with decreased levels of the proinflammatory cytokine TNF-*α* and increased levels of the anti-inflammatory cytokine IL-10. In addition, a decrease in hippocampal BDNF levels was found, suggesting a reduction in the ability to cope with prolonged stress (brain plasticity). 

Analyzing more in detail the effects of RS, we found that, following 7 days of this procedure, CORT elevation was significantly lower than on the first stimulation (day 1), suggesting an habituation of the system to the chronic procedure, as previously shown [42]. This effect, which appears to be mediated by limbic regions [8], is likely to have consequences for the functioning of a number of GC-sensitive systems, including the immune system. Indeed, reduced CORT levels could disinhibit immune function, leading to a proinflammatory response, as suggested by the increase in the levels of TNF-*α*. 

A number of evidence support the hypothesis that a moderate increase in the levels of proinflammatory cytokines, such as TNF-*α*, might result in an overall “priming effect” on the immune system, leading to better abilities to cope with further physiologic/stressful stimuli [21, 43–46]. Worth noticing, the response to stress observed does not only involve peripheral targets, but also extends to central mediators. In fact, after 7 days of RS, hippocampal BDNF protein levels showed a trend towards an increase, possibly reflecting a neuroprotective mechanism. By contrast, after 21 days of RS, BDNF levels were found to be decreased and this was associated to an augmented anti-inflammatory response by the immune system with increased IL-10 levels and a return of TNF-*α* to basal levels. It must be emphasized that while acute changes in BDNF levels might represent a coping response to stressful events, and thus being beneficial, prolonged exposure to stressors and increased allostatic load would lead to detrimental effects as reduced BDNF signaling in the adult brain may be involved in the pathophysiology of psychiatric disorders [47–49].

A fine regulation of apoptosis might positively affect optimal immune function. This is achieved by maintaining lymphocyte homeostasis by a continuous removal of cells that have been activated once they have served their function. Therefore, inappropriate induction of such a mechanism could result in a variety of pathological effects such as autoimmune diseases, while the maintenance of physiologically regulated levels of apoptosis might exert a beneficial/protective effect [50]. In this context, the observed decrease in apoptosis levels following 21 days of RS, suggests a long-term impairment of the immune system response.

Compared to RS, SS resulted in an overall lower response of the HPA axis as well as of the immune system. Differences were both quantitative and qualitative. In particular, no change in TNF-*α* could be detected, while an earlier increase in IL-10 was observed compared to RS, suggesting an anticipated anti-inflammatory reaction in response to this specific stressor. Differently from RS data, splenic apoptosis increased after 7 days of SS, suggesting that it might represent a reliable early stress-sensitive physiological marker.

Taken together, data from this study clearly indicate a differential role of psychophysical versus social and of brief versus prolonged stress on neuroendocrine and immune function suggesting that the quality and the extent of the stress period are crucial in determining individual neuroendocrine-immune responses to external challenges. In addition, and more intriguingly, all these peripheral responses were associated to specific changes in hippocampal BDNF levels. We hypothesize that this neurotrophin might represent a key modulator of neuro-immunoendocrine pathways, playing a pivotal role in the orchestration/maintenance of the brain and peripheral plasticity leading to optimal coping strategies to stressful events [8]. Future studies should employ pharmacological challenges aimed at investigating such interactions.

While this is a first attempt to mimic some of the qualitative and temporal features of “stress,” studies are ongoing to extend the range of mediators analyzed and the peripheral targets, to evaluate more extensively the role of acute versus chronic stressors on neuroendocrine and immune function. In addition a thorough characterization of the specific changes occurring in brain plasticity in other regions involved in neuroendocrine-immune integration will help elucidating the mechanisms underlying the beneficial/pathological effects of stress increasing the translational value of these studies.
